# BRAF^V600E^ mutation, BRAF-activated long non-coding RNA and miR-9 expression in papillary thyroid carcinoma, and their association with clinicopathological features

**DOI:** 10.1186/s12957-020-01923-7

**Published:** 2020-06-27

**Authors:** Chenlei Shi, Jia Cao, Tiefeng Shi, Meihua Liang, Chao Ding, Yichen Lv, Weifeng Zhang, Chuanle Li, Wenchao Gao, Gang Wu, Jianting Man

**Affiliations:** 1 The Fourth Department of General Surgery, the Second Affiliated Hospital, Harbin Medical University, 246 Xuefu Road, Harbin, 150001 Heilongjiang Province China; 2 The Department of Head and Neck Surgery, General Hospital of Heilongjiang Province Land Reclamation Bureau, Harbin, 150088 Heilongjiang Province China; 3 The Fourth Department of General Surgery, Harbin First Hospital Affiliated to Harbin Institute of Technology, Harbin, 150010 Heilongjiang Province China

**Keywords:** Papillary thyroid carcinoma, BRAF ^V600E^ mutation, BRAF-activated long non-coding RNA, miR-9

## Abstract

**Background:**

The incidence of thyroid cancer is increasing worldwide. This study investigated the association of B-type RAF kinase (BRAF)^V600E^ mutation status, the expression of BRAF-activated long non-coding RNA (BANCR) and microRNA miR-9, and the clinicopathological features of papillary thyroid carcinoma (PTC).

**Methods:**

Clinicopathological data for PTC patients (*n* = 51) diagnosed and treated between 2018 and 2019 were collected. Carcinoma and adjacent normal tissue samples were analyzed for the presence of the BRAF^V600E^ mutation and/or expression of BANCR and miR-9.

**Results:**

Larger tumor, higher rate of bilateral tumors and multifocality, extracapsular invasion, and lateral lymph node metastasis (LNM) were observed in PTC patients with BRAF ^V600E^ mutation. Patients with higher BANCR expression had a higher rate of extracapsular invasion and lateral LNM in carcinoma tissue and a lower frequency of bilateral tumors and multifocality in normal adjacent tissue. Patients with higher miR-9 expression had a lower rate of central and lateral LNM in carcinoma tissue and higher rates of bilateral tumor location and multifocality in normal adjacent tissue. Patients with BRAF^V600E^ mutation have a higher rate of BANCR overexpression and tended to have a lower rate of miR-9 overexpression (*P* = 0.057), and a negative association was observed between BANCR and miR-9 expression in carcinoma tissue.

**Conclusions:**

BRAF^V600E^ mutation and the BANCR and miR-9 expression were closely associated with the tumor size, bilateral tumor location, multifocality, extracapsular invasion, and lateral LNM. PTC patients with these clinicopathological characteristics, BRAFV600E mutation, and high BANCR expression and low miR-9 expression needed earlier surgical treatment and are recommended for total thyroidectomy in primary surgery for reducing the risk of recurrence. These findings provide new insight into the molecular basis for PTC and can inform strategies for the management of PTC.

## Introduction

Thyroid cancer is an endocrine malignancy and its incidence is increasing worldwide, especially in developed countries [1]. Papillary thyroid carcinoma (PTC) is the most common pathological subtype of thyroid cancer, accounting for 80–90% of cases [2]. PTC is thought to arise through interactions between genetic and environmental factors. The most common genetic changes in PTC are mutations in B-type RAF kinase (BRAF) of the RAS/BRAF/mitogen-activated protein kinase signaling pathway [3]; the most frequent mutation (95%) is BRAF^V600E^ [4, 5], which is associated with enhanced extrathyroid extension, lymph node metastasis (LNM), and advanced tumor stage [6–8]. However, this has not been corroborated by other studies [9], and therefore the relationship between clinicopathological characteristics of PTC and BRAF^V600E^ mutation remains unclear.

Long non-coding (lnc)RNAs are RNA molecules with a length greater than 200 nt that are transcribed from non-protein-coding sequences in the genome. LncRNAs play an important role in gene regulation at the epigenetic, transcriptional, and translation levels and in post-translational protein modification [10, 11], and have been implicated in processes such as tumorigenesis as well as tumor progression, metastasis, and recurrence [12–15]. For example, the lncRNA BRAF-activated long non-coding RNA (BANCR) promotes proliferation, inhibits apoptosis and G1 arrest, and stimulates autophagy in IHH-4 thyroid cancer cells [16]. Micro(mi)RNAs are small ncRNAs with length of about 22 nt that negatively regulate the expression of target genes at the post-transcriptional level by inducing the degradation or inhibition of the translation of mRNAs. The interaction between lncRNA and miRNAs has been shown to influence tumor development and progression [17]. The miRNA miR-9 regulates the growth of cancer cells, and BANCR and miR-9 mutually regulate by altering the activity of nuclear factor (NF)-κB in gastric cancer cells [18]. However, it is unclear how this is related to clinicopathological features in PTC patients. Although thyroid tumor diagnosis has been improved by high-frequency ultrasound [19], about 20% of PTC patients show recurrence and have poor prognosis due to distant metastasis [20]. Clarifying the mechanisms underlying PTC progression can lead to the development of more effective treatment strategies.

In this study, we investigated the relationships between the molecular features of PTC including BRAF^V600E^ mutation and BANCR and miR-9 expression, and clinicopathological characteristics of PTC patients. Our findings provide important insight into the molecular basis for pathophysiological changes leading to PTC progression.

### Patients and method

#### Patients

PTC patients (*n* = 51) who underwent thyroidectomy during the period from March 2018 to October 2019 at the Second Affiliated Hospital of Harbin Medical University were recruited. The study was approved by the Ethics Committee of the Second Affiliated Hospital of Harbin Medical University (no. ky2018-155) and was carried out in accordance with the principles of the Helsinki Declaration. Written informed consent was provided by all participants. The inclusion criteria were as follows: (1) preliminary diagnosis by preoperative palpation and color ultrasound confirmed by intraoperative rapid pathology and postoperative pathology detection; (2) no history of thyroid disease and not receiving thyroid-related medications; (3) no history of Graves’ disease; and (4) surgery performed by the same team of doctors. Patient data including sex, age, tumor location (uni-/bilateral), tumor size, multifocality, extracapsular invasion, extrathyroid extension, Hashimoto’s disease, LNM location (central or lateral), and tumor-node-metastasis (TNM) stage were collected. The clinicopathological classification was carried out according to (2010) American Joint Committee (AJCC) on Cancer 7th Edition. Carcinoma tissue and adjacent tissue (normal gland tissue 5 mm from the tumor edge) were collected and analyzed for the presence of the BRAF^V600E^ mutation and BANCR and miR-9 expression.

#### Detection of BRAF^V600E^ mutation

Detection of the BRAF^V600E^ mutation in carcinoma tissue was performed as previously described [21]. Genomic DNA was isolated using a commercial kit (AmoyDx FFPE DNA Kit; Amoy Diagnostics, Xiamen, China) according to the manufacturer’s instructions. DNA concentration was measured with an ultraviolet spectrophotometer; the optical density at 260 nm (OD_260_) and OD_280_ were 1.8 and 2.0, respectively. BRAF^V600E^ mutation status was determined using a kit (AmoyDx BRAF^V600E^ Mutation Detection Kit; Amoy Diagnostics) on a CFX96 real-time PCR detection system (Bio-Rad, Hercules, CA, USA). The sample was classified as positive or negative for the mutation if the carboxyfluorescein fluorescence signal Ct value was < 28 and ≥ 28, respectively.

#### Real-time quantitative RT-PCR

Carcinoma (*n* = 51) and adjacent normal tissue (*n* = 31) samples were used to detect expression levels of BANCR and miR-9. Total RNA was extracted by TRIpure and reverse transcribed to cDNA using a cDNA Synthesis kit (BioTeke, Beijing, China) for BANCR, and a miRNA First Strand Synthesis kit (Takara, Dalian, China) for miR-9. Real-time PCR was performed on an Exicycler 96 fluorescence quantitative instrument (Bioneer, Seoul, Korea) using the primers shown in Table 1. The expression levels of BANCR and miR-9 were determined with the comparative method (2^−∆∆Ct^) relative to those of the *β-actin* and *5S* genes, respectively.

**Table 1:** Primers used in qRT-PCR

Primer	Sequence (5′→3′)	Size of target fragment (bp)
BANCR-F	CCCCTGACCCTAAGGAAATA	150
BANCR-R	GAACTGGCAAGGCTCAAACT	
β-actin-F	CTTAGTTGCGTTACACCCTTTCTTG	156
β-actin-R	CTGTCACCTTCACCGTTCCAGTTT	
miR-9-5p-F	CGCCGCTCTTTGGTTATCTAG	63
miR-9-5p-R	GTGCAGGGTCCGAGGTATTC	
5S-F	TCTCGTCTGATCTCGGAAGC	125
5S-R	TGGTGCAGGGTCCGAGGTAT	

#### Statistical analysis

Statistical analyses were performed using SPSS v.13.01S (Beijing Stats Data Mining Co., Beijing, China). Data are presented as mean ± SD or as a percentage as appropriate. Differences between groups were analyzed with the independent samples *t* test for continuous variables, and with the *χ*^2^ test or Fisher’s exact tests for the categorical variables. *P* values were two-tailed, and *P* < 0.05 was considered significant.

## Results

### Clinicopathological characteristics and prognosis of PTC patients

A total of 51 PTC patients who underwent thyroid surgery were enrolled in the study. There were no patients with distant metastasis of lung or bone; 14 of them underwent unilateral thyroidectomy and central lymph node dissection, 20 patients underwent total thyroidectomy and bilateral lymph node dissection, and 17 patients underwent total thyroidectomy and bilateral lymph node dissection and unilateral neck lymph node dissection. There were 37 females and 14 males. The mean age was 41.7 ± 11.6 years (range 23–62 years). Bilateral localization was observed in 60.8% of patients. The mean tumor size was 1.7 ± 0.8 cm (range 0.9–4.3 cm). Multiple tumors were found in 70.6% of patients. Extracapsular invasion and extrathyroid extension were observed in 52.9% and 15.7 of patients, respectively, and 11.8% had Hashimoto’s disease. Central LNM was present in 62.7% of patients while central and lateral LNM were detected in 33.3% of patients. T1, T2, and T3 of TNM stage were observed in 51.0%, 33.3%, and 15.7 of patients, respectively (Table 2).

**Table 2:** Clinicopathological characteristics of 51 PTC patients

Characteristics	Patients (*n*)	Percent (%)
Sex		
Female	37	72.5
Male	14	27.5
Age (years)	41.7 ± 11.6	
≤ 45	32	62.7
> 45	19	37.3
Tumor location		
Unilateral	20	39.2
Bilateral	31	60.8
Tumor size (cm/mean)	1.7 ± 0.8	
Multifocality		
Single	15	29.4
Multiple (≥ 2)	36	70.6
Extracapsular invasion		
No	24	47.1
Yes	27	52.9
Extrathyroid extension		
No	43	84.3
Yes	8	15.7
Hashimoto’s disease		
No	45	88.2
Yes	6	11.8
Central LNM		
No	19	37.3
Yes	32	62.7
Lateral LNM		
No	34	66.7
Yes	17	33.3
TNM stage		
T1	26	51.0
T2	17	33.3
T3	8	15.7

*LNM* lymph node metastasis; *TNM* tumor-node-metastasis

### Differences in clinicopathological characteristics of patients according to BRAF^V600E^ mutation status and BANCR and miR-9 expression in carcinoma tissue

Sex, age, extrathyroid extension, co-occurrence of Hashimoto’s disease, and TNM stage were unrelated to BRAF^V600E^ mutation status and BANCR and miR-9 levels in carcinoma tissue. However, patients with the mutation had larger tumors, a higher frequency of bilateral tumor, multifocality, extracapsular invasion, and lateral LNM compared with those without the mutation. Patients with higher BANCR expression had a higher rate of extracapsular invasion and lateral LNM, and those with higher miR-9 expression had a lower rate of central and lateral LNM but no significant differences in other variables relative to patients with lower miR-9 expression (Table 3).

**Table 3:** Relationships between clinicopathological characteristics of PTC patients and BRAF^V600E^ mutation status and BANCR and miR-9 expression in carcinoma tissue (*n* = 51)

	BRAF^V600E^ mutation (*n*)		*P* value	BANCR (*n*)		*P* value	miR-9 (*n*)		*P* value
	Yes	No		> 0.01	≤ 0.01		> 0.01	≤ 0.01	
Sex			0.471			0.742			0.475
Female	27	10		26	11		20	17	
Male	12	2		9	5		6	8	
Age (years)			0.497			0.221			0.180
≤ 45	23	9		20	12		14	18	
> 45	16	3		15	4		12	7	
Tumor location			< 0.001			0.654			0.645
Unilateral	9	11		13	7		11	9	
Bilateral	30	1		22	9		15	16	
Tumor size (cm/mean)	1.8 ± 0.7	1.3 ± 0.4	0.002	1.7 ± 0.8	1.5 ± 0.6	0.501	1.6 ± 0.8	1.7 ± 0.7	0.756
Multifocality			0.003			0.510			0.104
Single	7	8		9	6		5	10	
Multiple (≥ 2)	32	4		26	10		21	15	
Extracapsular invasion			0.004			0.036			0.121
No	14	10		13	11		15	9	
Yes	25	2		22	5		11	16	
Extrathyroid extension			0.173			0.694			0.140
No	31	12		30	13		24	19	
Yes	8	0		5	3		2	6	
Hashimoto’s disease			0.616			0.363			0.419
No	35	10		32	13		24	21	
Yes	4	2		3	3		2	4	
Central LNM			0.101			0.202			0.012
No	12	7		12	6		14	5	
Yes	27	5		23	10		12	20	
Lateral LNM			0.042			0.033			0.029
No	23	11		20	14		21	13	
Yes	16	1		15	2		5	12	
TNM stage			0.110			0.470			0.291
T1	17	9		16	10		15	11	
T2	14	3		12	5		9	8	
T3	8	0		7	1		2	6	

*BANCR* BRAF-activated long non-coding RNA, *LNM* lymph node metastasis, *TNM* tumor-node-metastasis

### Relationship between clinicopathological characteristics and BANCR and miR-9 expression in normal adjacent tissue

Patients with higher BANCR expression in adjacent normal tissue had a lower frequency of bilateral tumors and multifocality, and a larger tumor as compared to those with lower BANCR levels. However, patients with elevated miR-9 level had higher rates of bilateral tumors and multifocality. There was no relationship between the other variables and the expression of BANCR and miR-9 (Table 4).

**Table 4:** Relationship between clinicopathological characteristics and BANCR and miR-9 expression in adjacent normal tissue (*n* = 31)

	BANCR (*n*)		*P* value	miR-9 (*n*)		*P* value
	> 0.01	≤ 0.01		> 0.01	≤ 0.01	
Sex			0.063			0.185
Female	1	21		18	4	
Male	3	6		5	4	
Age (years)			0.621			0.123
≤ 45	3	15		11	7	
> 45	1	12		12	1	
Tumor location			0.007			0.006
Unilateral	4	6		4	6	
Bilateral	0	21		19	2	
Tumor size (cm/mean)	2.7 ± 1.3	1.6 ± 0.8	0.031	1.7 ± 0.9	1.8 ± 1.1	0.804
Multifocality			0.007			0.006
Single	4	6		4	6	
Multiple (≥ 2)	0	21		19	2	
Extracapsular invasion			0.607			0.412
No	1	13		9	5	
Yes	3	14		14	3	
Extrathyroid extension			0.268			0.642
No	2	21		16	7	
Yes	2	6		7	1	
Hashimoto’s disease			0.561			0.298
No	4	21		17	8	
Yes	0	6		6	0	
Central LNM			0.295			0.66
No	0	9		6	3	
Yes	4	18		17	5	
Lateral LNM			0.268			0.178
No	2	21		19	4	
Yes	2	6		4	4	
TNM stage			0.490			0.640
T1	2	15		12	5	
T2	0	6		4	2	
T3	2	6		7	1	

*LNM* lymph node metastasis, *TNM* tumor-node-metastasis

### Relationship between BANCR and miR-9 expression and BRAF^V600E^ mutation status

Patients with BRAF^V600E^ mutation have a higher rate of BANCR overexpression and tended to have a lower rate of miR-9 overexpression (*P* = 0.057) (Table 5). PTC patients had higher and lower rates of elevated BANCR and miR-9 expression, respectively, in carcinoma vs. adjacent normal tissue, and there was a negative association that was observed between BANCR and miR-9 expression in carcinoma (Table 6).

**Table 5:** Relationship between BRAF^V600E^ mutation status and BANCR and miR-9 expression in carcinoma tissues (*n* = 51)

	BRAF^V600E^ mutation		*P* value
	Yes	No	
BANCR			0.033
≤ 0.01	9	7	
> 0.01	30	5	
miR-9			0.057
≤ 0.01	22	3	
> 0.01	17	9	

*BANCR* BRAF-activated long non-coding RNA

**Table 6:** BANCR and miR-9 expression in carcinoma and adjacent normal tissues

Different tissues	miR-9		*P* value	BANCR		*P* value
	> 0.01	≤ 0.01		> 0.01	≤ 0.01	
Carcinoma tissue (*n* = 51)	26	25	0.038	35	16	< 0.001
Adjacent tissue (*n* = 31)	23	8		4	27	
Carcinoma tissue						
BANCR						
> 0.01	14	21	0.020	–		
≤ 0.01	12	4				
Adjacent tissue						
BANCR			0.268			
> 0.01	2	2		–		
≤ 0.01	21	6				

*BANCR* BRAF-activated long non-coding RNA

## Discussion

Among malignant thyroid tumors, PTC is a common histopathological subtype [22, 23]. Given that imaging modalities are used for physical examination of thyroid, the diagnosis rate of thyroid tumors is increasing worldwide. However, this has not translated into a decrease in mortality rate, although it is possible that thyroid cancer is overdiagnosed and overtreated [1, 24]. Ito Y et al. [20] reported that up to 20% of patients show recurrence and have poor prognosis due to distant metastasis in PTC metastasis. Therefore, the excessive or inadequacy treatment on PTC is confusing. Thus, the biological characteristics of PTC require clarification for finding suitable PTC patients of surgery through analyzing the relationship between their clinicopathological features, BRAF^V600E^ mutation status, and BANCR and miR-9 expression.

The BRAF^V600E^ mutation is the most common genetic change in PTC patients and is not observed in normal thyroid tissue or benign lesions; therefore, we did not evaluate BRAF^V600E^ mutation status in normal tissue in the present study. We detected the BRAF^V600E^ mutation in the carcinoma tissue of 76.5% patients; although it was unrelated to sex, age, extrathyroid extension, co-occurrence of Hashimoto’s disease, and TNM stage, patients with the mutation had larger tumors, a higher frequency of bilateral tumor location, multifocality, extracapsular invasion, and lateral LNM compared with those without the mutation. Consistent with our findings, a meta-analysis of 32 studies and 6372 patients found that BRAF^V600E^ mutation was associated with several of the variables used in prognostic staging systems such as tumor size, multifocality, and LNM [25]. Another study indicated that extracapsular invasion is an indicator of distant metastasis and poor prognosis in patients with PTC [26]. Our results showed that patients with higher expressions of BRAF^V600E^ mutation and BANCR have higher rates of extracapsular invasion.

In the present study, patients with higher BANCR expression had a higher rate of lateral LNM, and those with higher miR-9 expression had a lower rate of central and lateral LNM. In addition, patients with the BRAF^V600E^ mutation had a higher frequency of lateral LNM compared with those without the mutation. Previous studies have mainly focused on LNM in the central region [27, 28] based on complete central neck dissection. However, more attention needs to be paid to LNM in the lateral region, not only the confirmed LN should be dissect, but also preventive LN dissection should be performed in the lateral region to prevent the omission and recurrence of tumor. In addition, BANCR and miR-9 expression in adjacent normal tissue was also associated with tumor location, size, and multifocality. Therefore, for patients with high-risk clinicopathological characteristics, surgical treatment can be relaxed from glandular lobectomy to total thyroidectomy as appropriate.

BANCR is a recently identified lncRNA activated by BRAF that plays an important role in the occurrence and progression of PTC. A qRT-PCR analysis revealed that BANCR is highly expressed in PTC tissue compared with normal tissue; it was also found to promote proliferation, inhibit cell apoptosis, alleviate G1 arrest, and stimulate autophagy in IHH-4 thyroid cancer cells [15]. In addition, the expression of miR-9 was downregulated in carcinoma compared with adjacent normal tissue in the present study. Consistent with our finding, Sondermann A et al. [29] observed the significantly downregulated expression of miR-9 in patients with recurrent PTC, which can be used as a prognostic indicator of PTC. Gu Y et al. [30] found that miR-9 may inhibit the activity of PTC cells and tumor growth by directly targeting the expression of BRAF in PTC. In the present study, patients with BRAF^V600E^ mutation have a higher rate of BANCR overexpression and tended to have a lower rate of miR-9 overexpression (*P* = 0.057), and there is a negative association were found between BANCR and miR-9 expression levels. One study reported that, in gastric cancer cells, downregulation of BANCR decreased NF-κB activity and inhibited cell proliferation while promoting apoptosis, whereas overexpression of NF-κB—a target of miR-9 that regulates cancer cell growth—and inhibition of miR-9 reversed these effects [18]. Given that BANCR is a lncRNA activated by BRAF, we speculate that BRAF^V600E^ mutation and BANCR and miR-9 expression together contribute to PTC development and progression. There were some limitations to this study such as the small size of the study population and the fact that BANCR and miR-9 expression was not evaluated in all adjacent tissues. Therefore, additional studies are needed to validate our findings. In addition, BRAF^V600E^ mutation can be determined by fine-needle aspiration cytology with the current hospital detection technology, while the preoperative sample size is not enough to determine the BANCR and miR-9 expression. The sample size obtained during the operation is enough for determining the BRAF^V600E^ mutation, BANCR, and miR-9 expression, but there is still a lack of rapid detection and analysis technology at present. Therefore, rapid gene detection before and during operation is not available. By expanding the number of retrospective studies to increase the evidence of clinical inquiry and the development of rapid detection technology, it is expected that the research results will be used in clinical practice in the future.

## Conclusions

In conclusion, our results demonstrate that the tumor size, bilateral tumor location, multifocality, extracapsular invasion, and lateral LNM are closely associated with BRAF^V600E^ mutation, and the BANCR and miR-9 expression, which could be as the high-risk clinicopathological characteristics. Particularly, PTC patients with high-risk clinicopathological characteristics, BRAFV600E mutation, and high BANCR expression and low miR-9 expression needed earlier surgical treatment, and are recommended for total thyroidectomy in primary surgery for reducing the risk of recurrence. These findings provide new insight into the molecular basis for PTC and can inform strategies for the management of PTC.

## Data Availability

The datasets generated and/or analyzed during the current study are available from the corresponding author on reasonable request.
